# Advances in Vitreoretinal Interface Disorders

**DOI:** 10.1155/2017/7612712

**Published:** 2017-07-02

**Authors:** Irini Chatziralli, Panagiotis Theodossiadis, Luke Nicholson, David Steel

**Affiliations:** ^1^2nd Department of Ophthalmology, Attikon Hospital, University of Athens, Athens, Greece; ^2^National Institute for Health Research, Moorfields Biomedical Research Centre and University College London Institute of Ophthalmology, London, UK; ^3^Sunderland Eye Infirmary, Queen Alexandra Road, Sunderland, UK; ^4^Institute of Genetic Medicine, Newcastle University, Newcastle upon Tyne, UK

In recent years, there have been a variety of advances in vitreoretinal interface (VRI) disorders. Multimodal imaging, such as spectral domain and swept source optical coherence tomography (OCT), allows for enhanced visualization of vitreous evolution and leads to a better understanding of VRI diseases in terms of both their diagnosis and management. However, the role of vitreous per se in these diseases has been neglected. Q. Ghadiali et al. tried to evaluate the impact of the vitreous body and the extent of its degeneration in eyes with vitreomacular traction (VMT) and macular hole (MH), showing that the eyes with VMT and MH demonstrated earlier stages of vitreous degeneration when compared to the control eyes, despite significantly greater age. The authors concluded that VRI diseases are more often associated with a formed vitreous and an intact premacular bursa. They hypothesized that the state of the vitreous itself may play a role in the aetiology of VMT, in addition to the strength of vitreoretinal adhesion, contrary to previous assumptions implicating degeneration of vitreous as a precipitating factor of VRI disease when in conjunction with abnormal vitreomacular separation.

Additionally, posterior vitreous detachment (PVD) can act as a protecting factor in cases with endogenous endophthalmitis. K. Umazume et al. retrospectively studied ten patients with endogenous endophthalmitis and found that despite an advanced age during the vitrectomy, PVD was absent in 80%, 50% of which showed retinal necrosis, supporting that the state of vitreous attachment may be related to the occurrence of endogenous endophthalmitis. In fact, the authors suggested that the contact between the retina and the vitreous body may be associated with severe disease and proposed that the gel-like vitreous body may play the role as a growth medium for the causative microorganisms.

On the other hand, there have also been many advances in surgical techniques for the treatment of VRI diseases. U. Soiberman et al. in a retrospective study of 74 eyes compared the outcome of MH surgery with that of ILM peeling using two different vital dyes, namely, membrane blue and membrane blue dual. They concluded that membrane blue dual led to better visual results probably due to better staining and lesser toxicity, while there was no difference in MH closure rate between the two dyes. Furthermore, A. Bedda et al. reported the anatomic and visual results of a new sutureless illuminated macular buckle designed for patients with the complex clinical situation of MH retinal detachment related to high myopia with an axial length of >30 mm. They achieved a MH closure rate of 40% postoperatively with a significant increase of visual acuity from 0.11 to 0.21 (decimal) without complications in the 20 studied eyes.

Nevertheless, although the majority of surgical techniques have been found to be safe and effective for VRI disease management, transient or permanent postoperative complications have been reported. K. Ohta et al. supported that the postoperative retinal nerve fiber layer (RNFL) was thicker in all but the nasal-inferior sector for at least 12 months after combined phacoemulsification and pars plana vitrectomy (PPV) with ILM peeling for patients with MH. This prolonged increase of the RNFL thickness may indicate damage or mild edema of the RNFL.

Finally, P. Stavrakas et al. in their retrospective study evaluated the anatomic and functional outcomes of patients with rhegmatogenous retinal detachment primarily treated with PPV based on the location of the retinal breaks. The authors found that there was no statistically significant difference between patients with superior and inferior breaks with regard to primary retinal reattachment, mean visual acuity change, and complication rate. Therefore, PPV alone can be used to treat uncomplicated rhegmatogenous retinal detachment irrespective to the location of the breaks, suggesting that inferior breaks do not constitute an independent risk factor for worse anatomic or functional outcome.

*Irini Chatziralli* *Irini Chatziralli* *Panagiotis Theodossiadis* *Panagiotis Theodossiadis* *Luke Nicholson* *Luke Nicholson* *David Steel* *David Steel*

